# Chronic Care Model Decision Support and Clinical Information Systems Interventions for People Living with HIV: A Systematic Review

**DOI:** 10.1007/s11606-012-2145-y

**Published:** 2012-07-13

**Authors:** Anjori Pasricha, Roo T. M. Deinstadt, David Moher, Amanda Killoran, Sean B. Rourke, Claire E. Kendall

**Affiliations:** 1 Faculty of Medicine, University of Ottawa, Ottawa, Canada; 2 C.T. Lamont Primary Health Care Research Centre, Élisabeth Bruyère Research Institute, Ottawa, Canada; 3 Ottawa Hospital Research Institute, Ottawa, Canada; 4 Department of Epidemiology and Community Medicine, University of Ottawa, Ottawa, Canada; 5 Centre for Public Health Excellence, National Institute for Health and Clinical Excellence, Manchester, UK; 6 Ontario HIV Treatment Network, Toronto, Canada; 7 Department of Psychiatry, University of Toronto, Toronto, Canada; 8 Centre for Research on Inner City Health in the Keenan Research Centre of the Li Ka Shing Knowledge Institute of St. Michael’s Hospital, Toronto, Ontario Canada; 9 Department of Family Medicine, University of Ottawa, Ottawa, Canada

**Keywords:** HIV/AIDS, chronic disease management, Chronic Care Model, decision support, clinical information systems, practice guidelines, systematic review

## Abstract

**BACKGROUND:**

The Chronic Care Model is an effective framework for improving chronic disease management. There is scarce literature describing this model for people living with HIV. Decision Support (DS) and Clinical Information Systems (CIS) are two components of this model that aim to improve care by changing health care provider behavior.

**OBJECTIVE:**

Our aim was to assess the effectiveness of DS and CIS interventions for individuals with HIV, through a systematic literature review.

**DESIGN:**

We performed systematic electronic searches from 1996 to February 2011 of the medical (E.g. Medline, EMBASE, CINAHL) and grey literature. Effectiveness was measured by the frequency of statistically significant outcome improvement. Data and key equity indicator extraction and synthesis was completed.

**PARTICIPANTS AND INTERVENTIONS:**

We included comparative studies of people living with HIV that examined the impact of DS or CIS interventions on outcomes.

**MAIN MEASURES:**

The following measures were assessed: outcome (immunological/virological, medical, psychosocial, economic measures) and health care process/performance measures.

**KEY RESULTS:**

Records were screened for relevance (n = 10,169), full-text copies of relevant studies were obtained (n = 123), and 16 studies were included in the review. Overall, 5/9 (55.6%) and 17/41 (41.5%) process measures and 5/12 (41.7%) and 3/9 (33.3%) outcome measures for DS and CIS interventions, respectively, were statistically significantly improved. DS–explicit mention of implementation of guidelines and CIS-reminders showed the most frequent improvement in outcomes. DS-only interventions were more effective than CIS-only interventions in improving both process and outcome measures. Clinical, statistical and methodological heterogeneity among studies precluded meta-analysis. Primary studies were methodologically weak and often included multifaceted interventions that made assessment of effectiveness challenging.

**CONCLUSIONS:**

Overall, DS and CIS interventions may modestly improve care for people living with HIV, having a greater impact on process measures compared to outcome measures. These interventions should be considered as part of strategies to improve HIV care through changing provider performance.

**Electronic supplementary material:**

The online version of this article (doi:10.1007/s11606-012-2145-y) contains supplementary material, which is available to authorized users.

## INTRODUCTION

While the global incidence of HIV infection has stabilized, the overall number of people living with HIV has steadily increased, as HIV treatments extend life.^1^ In the developed world, mortality from non-AIDS events now exceeds that of AIDS-defining opportunistic diseases in individuals receiving effective antiretroviral therapy (ART).^2^,^3^ There is an increasing role, particularly in high-resource settings, for shifting our approach to HIV care from one of tertiary/specialist care to one that includes the prevention and treatment of common diseases.^4^–^6^ As HIV infection moves into the realm of a chronic disease managed primarily in the ambulatory setting, it is important to understand how the principles of chronic disease management can be applied to this population.

The Chronic Care Model (CCM; Wagner Model)^7^ is a well-established framework for effective, evidence-based clinical and quality improvement in chronic disease management. CCM initiatives have become the foundation of patient care for ischemic heart disease,^8^,^9^ congestive heart failure,^10^–^12^ diabetes,^10^,^11^,^13^ asthma,^10^,^11^,^13^ chronic obstructive pulmonary disease (COPD),^14^ and depression,^10^ and have been shown to improve patient-reported health status and quality of life in primary care settings.^15^ However, literature on the application of the CCM framework, and its elements for HIV management in the primary care setting, is limited.

Decision Support (DS) and Clinical Information Systems (CIS) are two elements of the CCM that specifically target changing provider behavior to improve patient care. DS interventions, such as the distribution of educational materials, use of clinical practice guidelines, and case discussions, emphasize the integration of evidence-based guidelines into clinical practice. CIS interventions are based on establishing information systems to organize patient data in order to improve the delivery of care, such as by developing rosters of patients with certain conditions and providing reminders.^7^ Several systematic reviews have attempted to determine the effectiveness of these elements on patient care, although clinical, methodological and statistical heterogeneity of the included studies made data synthesis and generalizability difficult.^10^,^16^–^18^ As technology advances, DS and CIS interventions have become intertwined.^16^,^18^,^19^ In fact, computerization of decision support tools is likely an important feature contributing to their effectiveness.^13^,^19^ Some systematic reviews have examined the DS and CIS elements explicitly in the context of the CCM framework,^10^,^13^ in addition to many that have not directly identified these components, but have studied interventions that clearly fall under these categories.^17^–^21^ These reviews demonstrate that DS and CIS interventions are often successful,^10^,^13^,^17^–^22^ although the magnitude of effect may be modest^17^ and also more clearly improve provider performance than patient health measures.^10^,^13^,^18^

The purpose of this systematic review is to describe the application and effectiveness of DS and CIS interventions for persons living with HIV, and to identify the successful characteristics of these interventions.

## METHODS

### Protocol

A protocol for record eligibility was developed a priori. However, record screening began prior to the launch of PROSPERO, a prospective register of systematic review protocols.

### Eligibility Criteria

#### Population

Individuals known to be living with HIV with no restrictions based on age, gender, geography, setting, or transmission group.

#### Types of Interventions

Similar to the method of Zwar et al.,^13^ intervention strategies for people living with HIV were categorized according to the Effective Practice and Organization of Care (EPOC) taxonomy of interventions (http://epoc.cochrane.org/information-specific-epoc-reviews, accessed June 1, 2012), pertaining to the DS or CIS elements of the CCM. The EPOC taxonomy was used because of its focus on interventions designed to improve professional practice and delivery of health care, which fits with the scope of this review. This taxonomy allows mapping of the interventions to the elements of the CCM, which facilitated a descriptive categorization of the interventions.

#### Types of Comparison Groups

Studies without a comparison group were excluded. Comparators included usual care, another (non-CCM) intervention, or both.

#### Types of Assessment Measures

The effectiveness of interventions in this review was evaluated by assessing improvement in “outcome” measures and “healthcare process/provider performance” measures, as defined by Adair et al.^23^

### Primary Measures of Assessment

We identified a priori the following “outcome” measures for included studies:

1) Immunological or virological outcomes: CD4 count or viral load

2) Medical outcomes: mortality of patients, progression to AIDS, opportunistic infections and cancers, hospitalizations, functional status/disability, adherence to medication, and change in at-risk behaviors

3) Psychosocial outcomes: an outcome measure for quality of life or psychological health and well-being

5) Economic outcomes: information about healthcare utilization (length of stay, emergency department visits, visits to providers), costs of treating patients, and costs to patients of healthcare received

“Healthcare process/provider performance” measures were also assessed. While these were not selected a priori, they included any measures of processes that are assumed to improve patient care, such as:

1. Health care professional adherence to guidelines
2. Proportion of patients on antiretrovirals
3. Proportion of patients on indicated prophylaxis
4. Rates of screening for HIV-related illness
5. Provision of counseling
6. Rates of appropriate vaccination
7. Identification of at-risk behaviors
8. Patient or provider satisfaction with care

#### Types of Studies

Randomized clinical trials (RCTs), controlled clinical trials (CCTs), cohort studies, case-control studies, and controlled before and after designs were included.

### Information Sources

The comprehensive literature search strategy was informed by previous systematic reviews based on the CCM^13^ and the HIV/AIDS^5^ literature (See Appendix 1, available online), and was developed by an experienced medical librarian.

We searched the literature from 1996 (the advent of modern ART) to February 2011. First, we conducted a search of electronic databases that cover international literature in medical/health sciences, psychology, social sciences and social work (including, but not restricted to, MEDLINE, EMBASE, CINAHL, and Cochrane Library). Second, hand searching was performed, as needed, within reference lists of included studies. Third, we used an Internet search strategy of grey literature to identify other published and unpublished literature. Since some studies that were published in 1996 or later included data collected prior to 1996, a post-hoc decision was made to exclude these studies; as they were pre-ART, interventions and outcomes were not deemed relevant to current practice.

### Study selection

Records were combined into Distiller SR (http://systematic-review.net/), a web-based systematic review reference management software program. Next, the database was filtered for duplications to derive a unique set of records. We used five stages to review the articles:

#### Stage 1: Screening

The titles and abstracts of records identified by the search were screened using a checklist within Distiller SR to eliminate titles/topics that were not pertinent to the research question. Due to the large number of records to be screened, 40 records were first screened independently by two reviewers (CK, RD). The weighted kappa score between reviewers was 0.99, thus subsequent records were screened by only one reviewer. If there was any uncertainty regarding inclusion/exclusion, the article proceeded to Stage 2.

#### Stage 2: Verification of Eligibility Criteria

Using a priori study eligibility criteria, full-text copies of the potentially eligible studies were assessed by two reviewers (CK, AP, RD) to determine whether they fulfilled the inclusion criteria. The third reviewer resolved any disagreements.

#### Stage 3: Data Extraction

Data were extracted from each included study using a standardized data extraction form. The form was developed based on relevant literature in the area and derived by one reviewer (RD). While the data extraction form was not formally piloted and compared between reviewers, a small sample of studies was used by one reviewer (AP) to ensure that the form was comprehensive and easy to understand.^24^ Data extracted included participant demographics, study design, description of the DS or CIS interventions, and measures of assessment.

#### Stage 4: Data Synthesis

Summary tables containing all information abstracted from eligible studies were created. Comparability of studies was assessed by careful review of the population, interventions, comparators, and outcomes by one reviewer (AP). If two or more comparable studies were identified, a pooled estimate of effect was calculated in a meta-analysis to explore the effectiveness of the intervention. For studies looking at dichotomous data, relative risk (RR), odds ratio (OR), and/or risk difference are provided where reported. For continuous data, means and standard deviations or standard errors were extracted where available. For studies that were not comparable in terms of population, intervention, comparator, or assessment measures, a qualitative summary is provided. Outcomes were deemed significant if the 95% confidence interval (CI) of their effect estimate did not include unity. Where CIs were not reported, we used a p-value of ≤ 0.05 (comparing intervention and control groups) as indicating statistical significance. We also described the improvement across studies by the category of outcome and by taxonomy of DS or CIS interventions.

### Consideration of Equity in Data Extraction and Synthesis^25^

It is likely that the success of DS and CIS interventions aiming to improve health and health outcomes is affected by the social determinants of health, and the context in which the interventions are implemented. During data collection, information was collected on the study patient populations across those PROGRESS + dimensions most likely to contribute to inequities (Appendix 2, available online), as well as the transmission risk group which is relevant to HIV care.^18^,^23^,^35^,^42^

#### Stage 5: Risk of Bias

Each included study was assessed by one reviewer (AP) for methodological quality, using standardized risk of bias checklists. The Cochrane Collaboration’s tool for assessing risk of bias was used for RCTs, clinical controlled trials, and comparison before and after studies. The Newcastle-Ottawa scales were used for cohort studies and case control studies. As there are no instruments validated for non-comparative studies, no tool was used to assess risk of bias. Summary figures were used to depict risk of bias in included studies.^24^

## RESULTS

**Figure 1.**
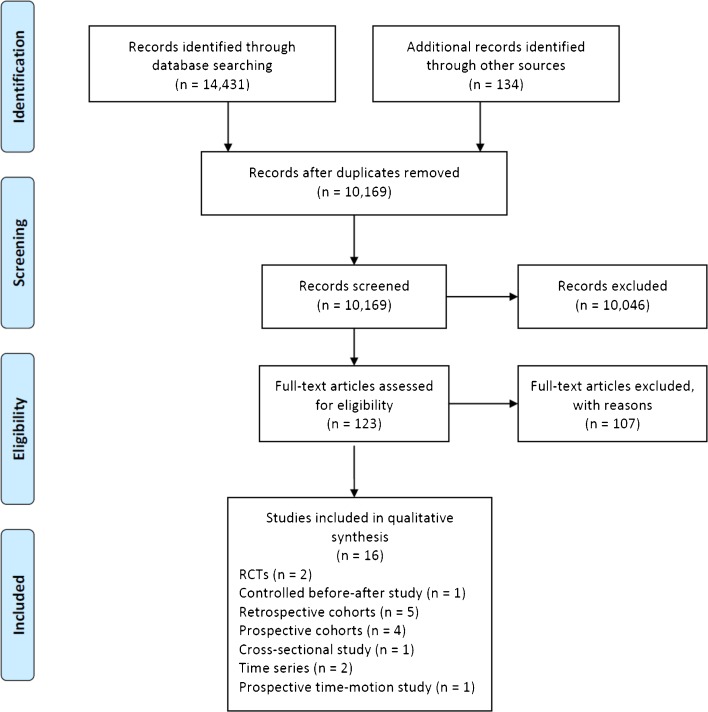
Flow Diagram of Included Studies.

We identified a total of 10,169 records (Figure 1). Our review process eliminated 10,046 records. Two articles were irretrievable, following extensive searches conducted by two medical librarians. Sixteen articles remained and were included.

### Participants of Included Studies

The total number of HIV positive patients included in this review is 29,897 across 13 studies. Two studies^26^,^27^ compared groups based on clinic/providers without reporting numbers of patients, and one^28^ did not report the total number of patients in the study. Of the studies reporting patients by gender, all but one study that examined cervical screening^29^ comprised a sample of mostly males. The age range of patients in all but one pediatric study was under 50 years.

### Settings of Included Studies

Ten studies were conducted in the USA,^2^,^26^,^29^–^36^ three in the UK/Europe,^37^–^39^ and three in sub-Saharan Africa.^27^,^28^,^40^ All studies examined care in the ambulatory setting.

### Design of Included Studies

Thirteen of the 16 studies (81.3%) were observational in design. Further details on the study designs can be found in Figure 1 and Table 1.

**Table 1:** Summary of Included Studies

Study (year)	Study design	Country	Number of centers	Sample size (CCM Intervention/Control)	Intervention	Measures of performance reported (Effective^*^/Total (%))	
						Process Measures	Outcome Measures
Bucher (2010)	RCT	Switzerland	7	Physicians randomized (57/60)	CIS– Audit/feedback	0/4	–
				Patients randomized (1634/1632)			
Pyne (2011)	RCT	U.S.A	3	138/138	DS– Communication and case discussion	–	2/8 (25%)
							Medical: 0/4
							Psych: 2/4 (50.0%)
Landon (2004)	CBA	U.S.A	44 intervention/25 control clinics	6406/3580	CIS– Presence of quality monitoring	0/6	0/2
							Imm/Vir: 0/1
							Economic: 0/1
Fonquernie (2010)	PCS	France	1	1717/1717	DS– Explicit mention of implementation of guidelines CIS– Reminders, Change in medical records systems	4/8 (50%)	2/2 (100%)
							Medical: 1/1 (100%)
							Psych: 1/1 (100%)
Gardner (2008)	PCS	U.S.A	17	1091/1091	DS– Educational meetings	1/1 (100%)	1/1 (100%)
					CIS– Audit/feedback		Medical: 1/1 (100%)
Horswell (2008)	PCS	U.S.A	8	3708/3708	CIS– Reminders, Change in medical records systems	2/6 (33.3%)	1/4 (25%)
							Imm/vir: 1/2 (50%)
							Medical: 0/1
							Economic: 0/1
Kitahata (2003)	PCS	U.S.A	1	1204/1204	CIS– Reminders	4/12 (33.3%)	–
Belperio (2009)	RCS	U.S.A	Not reported	7220/7220	DS– Distribution of educational materials	1/4 (25%)	–
Brown (2002)	RCS	U.S.A	1	73/73	CIS– Reminders	5/5 (100%)	–
Ma (2010)	RCS	U.S.A	1	75/75	DS– Communication and case discussion	–	3/4 (75%)
							Imm/Vir: 2/3 (66.7%)
							Medical: 1/1 (100%)
Natha (2008)	RCS	U.K.	1	100/100	DS– Explicit mention of implementation of guidelines	4/5 (80%)	–
Shuter (2003)	RCS	U.S.A	1	1026/1026	CIS– Audit/feedback	1/1 (100%)	–
Magnus (2009)	SCS	U.S.A	8	Not reported	CIS– Reminders, Change in medical records systems	1/3 (33.3%)	–
Morris (2009)	TS	Zambia	19	Not reported	DS– Educational meetings	0/3	–
					CIS– Audit/feedback, Presence of quality monitoring		
Youngleson (2010)	TS	South Africa	17	Not reported	CIS– Presence of quality monitoring	4/4 (100%)	–
Were (2010)	TM	Uganda	1	94/88	CIS– Reminders, Change in medical records systems	–	2/3 (66.7%)
							Economic: 2/3
**Total**						27/62 (43.5%)	11/24 (45.8%)
							Imm/Vir: 3/6 (50.0%)
							Medical: 3/8 (37/5%)
							Psych: 3/5 (60.0%)
							Economic: 2/5 (40.0%)

^*^Effective: Process or outcome measures showing statistically significant improvement

RCT: Randomized Clinical Trial, CCT: Controlled Clinical Trial, PCS or RCS: Prospective or Retrospective Cohort Study, CBA: Controlled before and after design, SCS: Serial Cross-sectional Study, TS: Time series (Interrupted), TM: Time-motion Study (Prospective)

Psych: Psychosocial measures, Imm/Vir: Immunological/Virological measures

### Risk of Bias and Methodological Quality of Included Studies

Risk of bias for included RCTs and controlled before and after studies is presented in Appendix 3, Table 1 (available online). Both RCTs used an appropriate method for randomization, although it was unclear whether allocation concealment was adequate in either. The risk of bias for cohort, time-motion, and prospective time-series studies was assessed using the Newcastle-Ottawa scale, and results are presented in Appendix 3, available online, Table 2. Six of these studies did not provide adequate information regarding follow-up of patients,^26^,^27^,^31^,^38^–^40^ and another seven did not provide any details regarding the control of important confounding variables between the intervention and control groups.^26^,^27^,^31^,^34^,^36^,^38^,^39^

### Effects of DS and CIS Interventions

Nine out of 16 (56%) studies assessed only health care process/performance measures, while three studies (19%) examined only outcome measures. The remaining four out of 16 studies assessed both types of measures. There was significant heterogeneity of study design, patient populations, types of interventions and outcomes examined among the studies (Table 1). Thus, a narrative summary of the evidence is presented in the summary of included studies in Appendix 4 (available online). While heterogeneity makes comparisons across studies impossible, we have included any effect estimates reported in the included studies for maximum transparency.

#### Studies Evaluating only Decision Support Interventions

There were four studies that looked at DS-only interventions: one assessed the distribution of educational materials,^34^ one included explicit mention of implementation of guidelines into practice,^37^ and two involved communication and case discussion^35^,^36^ interventions (Table 1). Two studies reported process measures,^34^,^37^ and found 5/9 (55.6%) of measures were significantly improved. Two studies reported outcome measures,^35^,^36^ and found 5/12 (41.7%) of measures were significantly improved (2/3 immunological/virological, 1/5 medical, 2/4 psychosocial). The DS intervention reporting the highest proportion of significantly improved outcomes was the explicit mention of implementation of guidelines, with improvement in four out of five (80%) healthcare process/performance measures.

#### Studies Evaluating only Clinical Information Systems Interventions

There were nine included studies that looked at CIS-only interventions: two examined reminders,^30^,^41^ two examined audit and feedback,^29^,^33^,^38^ two examined presence of quality monitoring^28^,^31^ and three studies examined both reminders and changes in medical records systems.^26^,^32^,^40^ Of the eight studies reporting process measures,^26^,^28^–^32^,^38^,^41^ 17/41 (41.5%) were statistically significantly improved. For the three studies reporting outcome measures,^31^,^32^,^40^ 3/9 (33.3%) of outcomes were improved, and there was no discernable pattern regarding the type of outcome measures that improved or not. With a total of 9/17 (52.9%) improved outcomes, the use of reminders was the most effective CIS intervention.

#### Studies Evaluating Interventions Combining Decision Support and Clinical Information Systems Interventions

Three studies^27^,^33^,^39^ implemented both DS and CIS interventions. One study (Gardner et al.^33^) used primarily provider training (DS-educational meetings, along with CIS-audit and feedback at one site only). Another (Morris et al.^27^) performed a complex, task-shifting traineeship that was primarily DS-educational meetings, but included CIS-quality monitoring and CIS-audit and feedback. The third study (Fonquernie et al.^39^) combined primarily CIS interventions (CIS-reminders and CIS-changes to medical records systems) with DS-explicit implementation of guidelines. Two of these studies assessed both process and outcome measures, while one looked at process measures only. For process measures, the findings varied with a total of 5/12 (41.7%) of measures improved; one study reported 4/8 (50%) measures as having improved, another reporting 1/1 measure as improved, and the third reporting no improvement in process measures (0/3). All outcome measures assessed in these studies showed statistically significant improvement (3/3; two medical and one psychosocial).

#### Study Features Relating to Outcome Improvement

As heterogeneity precluded meta-analysis, we examined whether study design, number of participants or study setting was associated with effectiveness. In the two RCTs, 0/4 of process measures and 2/8 (25%) of outcome measures were improved, compared to 27/58 (46.6%) and 9/16 (56.3%) of process and outcome measures, respectively, in the observational studies. In studies with >1000 patients, 13/42 (30.1%) of process measures and 4/9 (44.4%) of outcome measures were improved, compared to 16/23 (69.6%) and 5/12% (41.7%) of process and outcome measures in smaller (<1000 patients) studies. In addition, for the three largest studies with several thousand participants each,^31^,^32^,^34^ only 3/16 (18.8%) of process measures and 1/6 (16.7%) of outcome measures were significantly improved. Finally, the three studies conducted in Africa^27^,^28^,^40^ versus Europe or North America were all too different to assess the impact of setting on the effectiveness of interventions.

### Other Considerations

Equity indicators were poorly reported overall, and details are outlined in Appendix 5 (available online). When reported, these indicators did not provide any significant insights into the populations for which the interventions were more effective compared to others.

## DISCUSSION

Our review aimed to determine the effectiveness of Decision Support and Clinical Information Systems interventions in improving the care of persons with HIV, through the influence of provider behavior. There was considerable heterogeneity between study populations, settings, types of interventions, types of measures, and methodological quality of the included studies. This heterogeneity is consistent with existing literature examining the effectiveness of the Chronic Care Model^10^,^16^–^18^ for other conditions. While it is difficult to discern an overall pattern from the results, several observations can be made.

DS interventions were more likely than CIS interventions to improve process and outcome measures. The DS intervention most likely to be effective was the explicit implementation of guidelines, although this intervention on its own was implemented in only one study.^37^ This is similar to findings by Zwar et al.,^13^ although these authors also found DS-distribution of educational materials to be more effective in other conditions than we did for HIV, and found DS-educational meetings to be effective, while our review did not identify any studies examining this intervention. The most frequently effective CIS intervention in our review was the implementation of provider reminders, which was reported to improve health care process/provider performance outcomes in two studies.^30^,^41^ The least effective CIS intervention was audit and feedback. Identifying reminders as highly effective is consistent with Davis et al.^20^ and with Garg et al.,^18^ who found reminders to be the most effective form of computerized decision support. However, this finding is in contrast to Zwar et al.^13^ who found CIS-audit and feedback to be more effective for other conditions than we did for HIV. The magnitude of effect, which varies among studies, is consistent with previous work defining improvements as significant but of modest effect size.^10^,^17^,^22^

Studies assessing a combination of DS/CIS interventions were less likely than DS-only and equally likely to CIS-only interventions to improve process measures, and more likely to report improved outcome measures, although this number of studies is too small for definitive comparison. These findings add to the debate in the literature regarding whether the CCM must be implemented into practice as a whole, or whether individual elements can improve chronic disease management. The reality is that most interventions are multifaceted,^10^,^11^,^13^,^16^,^17^,^21^ and while some reviews have found that more than one intervention within or across CCM elements may be more effective than a single intervention,^11^,^16^,^20^ others have not.^10^,^13^,^17^

The number of RCTs was small and their risk of bias high, and these studies showed less effectiveness than those in observational study designs. In addition, larger studies were less likely to result in improved measures than smaller studies. Publication bias may have resulted in smaller, negative trials not being published.

Our work is consistent with previous reviews describing that DS and CIS interventions improve care, although we found a smaller proportion of improved outcomes than other reviews.^17^,^18^ Certain features of DS and CIS interventions improve their effectiveness, including the intensity of the intervention;^16^,^22^ the presence of a preceding practice-specific needs assessment;^20^ the involvement of the providers in the development of guidelines,^13^ process integration,^13^,^19^,^20^ the use of computer-based systems;^16^,^19^ and the provision of the reminder to patients themselves.^13^ The studies included in this review do not provide sufficient detail to extrapolate these potentially successful features to the setting of HIV/AIDS care. As with previous literature, we found that, overall, DS and CIS interventions improve processes of care, but that these improvements do not clearly translate into improved patient outcomes.^10^,^13^,^18^,^22^,^42^

This systematic review has several important limitations. First, our summary and synthesis is limited by the methods used in the primary studies, most of which were of observational design and lacked detailed descriptions of the interventions, a common pitfall of health care research.^43^ Second, studies included in the review did not monitor the long-term impact of the interventions that would make them more applicable for use in routine clinical practice.^44^ Third, there were a limited number of studies examining each intervention and outcome. The clinical heterogeneity of these interventions, outcomes and populations precluded our ability to perform a meta-analysis. Finally, many of the studies included multiple interventions, often across both DS and CIS categories, or were multifaceted to the extent that attributing effectiveness to one component of the intervention was difficult.^45^

Overall, DS and CIS interventions may modestly improve care for people living with HIV, with process measures more likely to be improved than definitive outcome measures. However, the limitations of the included studies precluded us from delineating the ingredients that influence effectiveness. Future studies aiming to change provider behavior through the implementation of DS and CIS interventions for people with HIV should use experimental, rather than observational, methodologies in larger samples of patients. Interventions should be described in detail^43^ and attention paid to known facilitating characteristics of effective DS and CIS interventions, especially when implemented in a broader quality improvement or CCM initiative. In addition, the importance of equity indicators in the design, implementation, and evaluation of interventions should be considered and reported.^25^ Finally, in addition to intermediate measures of process of care, studies should be extended to determine whether sustained and clinically significant differences can be found in patient-level outcomes.

## Electronic supplementary materials

Below is the link to the electronic supplementary material.

ESM 1 (DOCX 1478 kb)

## Abbreviations

ART: Antiretroviral therapy
CCM: Chronic Care Model
CIS: Clinical Information Systems
DS: Decision Support
EPOC: Effective Practice and Organization of Care
HIV/AIDS: Human Immunodeficiency virus/Acquired Immune Deficiency Syndrome
