# Supplementary material for: Chronic Care Model Decision Support and Clinical Information Systems Interventions for People Living with HIV: A Systematic Review
Source: J Gen Intern Med. 2012 Jul 13;28(1):127–35. doi: 10.1007/s11606-012-2145-y (PMC3539016; doi:10.1007/s11606-012-2145-y)
Supplement: Supplementary file 1 — (DOCX 1478 kb) [file 11606_2012_2145_MOESM1_ESM.docx]

**Chronic Care Model Decision Support and Clinical Information Systems interventions for people living with HIV: A Systematic Review**

**Appendices**

**Appendix 1: Literature search**

**CCM—SEARCH STRATEGIES**

**1579+2163+2681+1941**

**MEDLINE**

Database: Ovid MEDLINE(R) <1948 to February week 3 2011>, Ovid MEDLINE(R) Daily Update <February 23, 2011>, Ovid MEDLINE(R) In-Process & Other Non-Indexed Citations <February 23, 2011>
Search Strategy:
--------------------------------------------------------------------------------
1     Chronic Disease/th [Therapy] (2922)
2     "Quality of Health Care"/ (47095)
3     Disease Management/ (7304)
4     Models, Organizational/ (12247)
5     Pamphlets/ (2691)
6     Practice guidelines as topic/ (59661)
7     exp Patient participation/ (14526)
8     Decision support techniques/ (8528)
9     Reminder systems/ (1590)
10     (Chronic care model or Decision support or Practice guideline$ or Decision aid$ or Audiovisual aid$ or Audiovisual material$ or Education$ meeting$ or Opinion leader$ or Reminder$ or pamphlet$ or brochure$).tw. (24011)
11     audiovisual aids/ (5772)
12     Decision Support Systems, Clinical/ (3407)
13     quality assurance, health care/ (42480)
14     exp Information Systems/ (128478)
15     exp Decision Making, Computer-Assisted/ (68624)
16     exp Management Information Systems/ (33229)
17     exp Ambulatory Care Information Systems/ (1128)
18     REIMBURSEMENT, INCENTIVE/ (2045)
19     exp Registries/ (41731)
20     exp Clinical audit/ (16246)
21     Focus groups/ (11173)
22     exp mass media/ (34813)
23     ((patient$ or practic$) adj guideline$).tw. (11583)
24     (recall adj2 system$).tw. (323)
25     "audit and feedback".tw. (317)
26     focus group$.tw. (13906)
27     mass media.tw. (2775)
28     television.tw. (8421)
29     radio.tw. (24105)
30     newspaper$.tw. (3499)
31     (poster or posters).tw. (2582)
32     or/1-31 (520741)
33     HIV long-term survivors/ (426)
34     exp Anti-HIV agents/ (42385)
35     Antiretroviral Therapy, Highly Active/ (12715)
36     exp HIV infections/ (194941)
37     (human immunodeficiency or hiv or acquired immunodeficiency syndrome or acquired immune deficiency syndrome or aids or HIV or PHA or PWHA or PLWA or PLWHA).tw. (271100)
38     or/33-37 (304312)
39     32 and 38 (11326)
40     limit 39 to yr=1996-2011 (8551)
41     randomized controlled trial.pt. (299889)
42     controlled clinical trial.pt. (81742)
43     randomized.ti,ab. (230984)
44     intervention studies/ (4579)
45     comparative study.pt. (1504645)
46     placebo.tw. (129175)
47     drug therapy.sh. (29075)
48     randomly.tw. (160052)
49     trial.tw. (268752)
50     groups.tw. (1077448)
51     exp case-control studies/ (488466)
52     exp cohort studies/ (1066119)
53     evaluation studies/ (143840)
54     (case-control or cohort study or cohort studies or controlled study or controlled studies or controlled trial or controlled trials or controlled clinical trial$ or comparative study or comparative trial$ or comparative studies or prospective trial* or prospective studies or longitudinal study or longitudinal studies or longitudinal trial$).tw. (341305)
55     experiment$.tw. (1164728)
56     intervention?.tw. (370589)
57     evaluat$.tw. (1672942)
58     effect?.tw. (3110036)
59     change$.tw. (1702474)
60     impact.tw. (333155)
61     or/41-60 (8031437)
62     40 and 61 (4977)
63     animal/ not human/ (3453622)
64     62 not 63 (4958)

**EMBASE**

Database: EMBASE <1996 to 2011 Week 07>
Search Strategy:
--------------------------------------------------------------------------------
1     health care quality/ or exp practice guideline/ (322744)
2     exp patient participation/ (9469)
3     Disease Management/ (5653)
4     exp medical decision making/ (51775)
5     exp information system/ (65231)
6     mass medium/ (8811)
7     exp register/ (27580)
8     reimbursement/ (19362)
9     (Chronic care model or Decision support or Practice guideline$ or Decision aid$ or Audiovisual aid$ or Audiovisual material$ or Education$ meeting$ or Opinion leader$ or Reminder$ or pamphlet$ or brochure$).tw. (23990)
10     ((patient$ or practic$) adj guideline$).tw. (12570)
11     (recall adj2 system$).tw. (202)
12     "audit and feedback".tw. (362)
13     mass media.tw. (1861)
14     television.tw. (4795)
15     newspaper$.tw. (2839)
16     (poster or posters).tw. (2929)
17     exp Human immunodeficiency virus/ (74099)
18     exp acquired immune deficiency syndrome/ (56865)
19     highly active antiretroviral therapy/ (20989)
20     anti human immunodeficiency virus agent/ (11139)
21     (human immunodeficiency or hiv or acquired immunodeficiency syndrome or acquired immune deficiency syndrome or aids).tw. (195635)
22     or/17-21 (219860)
23     Randomized Controlled Trial/ (241458)
24     (randomised or randomized or random$ allocat$).tw. (289507)
25     experiment$.tw. (761627)
26     impact.tw. (341718)
27     intervention?.tw. (365485)
28     chang$.tw. (1210937)
29     evaluat$.tw. (1432285)
30     effect?.tw. (2074318)
31     compar$.tw. (2159635)
32     exp screening/ (223071)
33     exp prevention/ (469650)
34     or/23-31 (5360158)
35     or/1-16 (494639)
36     22 and 34 and 35 (5645)
37     limit 36 to human (5038)

**PsycINFO**

Database: PsycINFO <1967 to February Week 3 2011>
Search Strategy:
--------------------------------------------------------------------------------
1     decision making/ or exp decision support systems/ (34591)
2     exp "quality of care"/ (6143)
3     exp treatment guidelines/ (2960)
4     disease management/ (2014)
5     client participation/ (1034)
6     exp information systems/ (18109)
7     exp Clinical Audits/ (133)
8     exp mass media/ (23338)
9     (Chronic care model or Decision support or Practice guideline$ or Decision aid$ or Audiovisual aid$ or Audiovisual material$ or Education$ meeting$ or Opinion leader$ or Reminder$ or pamphlet$ or brochure$).tw. (8465)
10     educational audiovisual aids/ (435)
11     ((patient$ or practic$) adj guideline$).tw. (2650)
12     (recall adj2 system$).tw. (76)
13     "audit and feedback".tw. (50)
14     focus group$.tw. (12586)
15     mass media.tw. (3533)
16     television.tw. (9391)
17     radio.tw. (2772)
18     newspaper$.tw. (4442)
19     (poster or posters).tw. (1106)
20     or/1-19 (114453)
21     exp hiv/ (24833)
22     (human immunodeficiency or hiv or acquired immunodeficiency syndrome or acquired immune deficiency syndrome or aids).tw. (38367)
23     21 or 22 (38429)
24     20 and 23 (3270)
25     experimental design/ or exp clinical trials/ or exp followup studies/ or exp quasi experimental methods/ (24425)
26     experiment$.tw. (229862)
27     impact.tw. (142294)
28     intervention?.tw. (170276)
29     change$.tw. (318535)
30     evaluat$.tw. (272343)
31     effect?.tw. (574966)
32     or/25-31 (1247602)
33     24 and 32 (1837)
34     exp animals/ (217889)
35     33 not 34 (1837)
36     limit 35 to yr=1996-2011 (1583)

**COCHRANE REGISTER OF CONTROLLED TRIALS**

Database: EBM Reviews - Cochrane Central Register of Controlled Trials <1st Quarter 2011>
Search Strategy:
--------------------------------------------------------------------------------
1     Chronic Disease/th [Therapy] (118)
2     "Quality of Health Care"/ (432)
3     Disease Management/ (239)
4     Models, Organizational/ (93)
5     Pamphlets/ (466)
6     Practice guidelines as topic/ (765)
7     exp Patient participation/ (559)
8     Decision support techniques/ (226)
9     Reminder systems/ (375)
10     (Chronic care model or Decision support or Practice guideline$ or Decision aid$ or Audiovisual aid$ or Audiovisual material$ or Education$ meeting$ or Opinion leader$ or Reminder$ or pamphlet$ or brochure$).tw. (2030)
11     audiovisual aids/ (204)
12     Decision Support Systems, Clinical/ (126)
13     quality assurance, health care/ (425)
14     exp Information Systems/ (1030)
15     exp Decision Making, Computer-Assisted/ (2288)
16     exp Management Information Systems/ (312)
17     exp Ambulatory Care Information Systems/ (22)
18     REIMBURSEMENT, INCENTIVE/ (23)
19     exp Registries/ (331)
20     exp Clinical audit/ (215)
21     Focus groups/ (186)
22     exp mass media/ (1141)
23     ((patient$ or practic$) adj guideline$).tw. (401)
24     (recall adj2 system$).tw. (35)
25     "audit and feedback".tw. (73)
26     focus group$.tw. (299)
27     mass media.tw. (108)
28     television.tw. (319)
29     radio.tw. (688)
30     newspaper$.tw. (183)
31     (poster or posters).tw. (312)
32     or/1-31 (10731)
33     HIV long-term survivors/ (1)
34     exp Anti-HIV agents/ (2564)
35     Antiretroviral Therapy, Highly Active/ (607)
36     exp HIV infections/ (5337)
37     (human immunodeficiency or hiv or acquired immunodeficiency syndrome or acquired immune deficiency syndrome or aids or HIV or PHA or PWHA or PLWA or PLWHA).tw. (8251)
38     or/33-37 (8905)
39     32 and 38 (297)
40     limit 39 to yr=1996-2011 (245)
41     randomized controlled trial.pt. (288482)
42     controlled clinical trial.pt. (79737)
43     randomized.ti,ab. (182584)
44     intervention studies/ (1298)
45     comparative study.pt. (123192)
46     placebo.tw. (111246)
47     drug therapy.sh. (283)
48     randomly.tw. (77956)
49     trial.tw. (155162)
50     groups.tw. (158832)
51     exp case-control studies/ (6322)
52     exp cohort studies/ (84870)
53     evaluation studies/ (0)
54     (case-control or cohort study or cohort studies or controlled study or controlled studies or controlled trial or controlled trials or controlled clinical trial$ or comparative study or comparative trial$ or comparative studies or prospective trial* or prospective studies or longitudinal study or longitudinal studies or longitudinal trial$).tw. (94876)
55     experiment$.tw. (28495)
56     intervention?.tw. (51495)
57     evaluat$.tw. (127602)
58     effect?.tw. (248893)
59     change$.tw. (93698)
60     impact.tw. (19702)
61     or/41-60 (525441)
62     40 and 61 (244)
63     animal/ not human/ (0)
64     62 not 63 (244)

**SOCIOLOGICAL ABSTRACTS, SOCIAL SCIENCES ABSTRACTS**

KW=((hiv or aids or acquired immunodeficiency syndrome) or (human
immunodeficiency virus) or (acquired immune deficiency syndrome)) and
KW=((decision support or decision making or practice guideline* or
patient guideline* or decision aid* or audiovisual aid* or audiovisual
material* or reminder* or recall system*) or (information systems or mass
media or education*  meeting* or opinion leader* or pamphlet* or
brochure*) or (television or radio or poster or posters or newspaper* or
chronic care model or client participation or quality monitoring or
"audit and feedback" or registries or registry)) and KW=((random* or
compar* or intervention* or effect*) or (change* or controlled))

**CINAHL**

**Appendix 2: List of Equity Indicators**

**PROGRESS+ equity indicators:**

1. Place of residence

2. Race/ethnicity/culture

3. Occupation

4. Gender

5. Religion

6. Education

7. Socioeconomic status

8. Social capital and networks

9. Disability

10. Sexual orientation

11. Age

12. Transmission risk group (not a PROGRESS+ but relevant to HIV/AIDS)

**Appendix 3: Risk of Bias**

**Table 1: Risk of Bias – Randomized Controlled Trials and Controlled Clinical Trial (The Cochrane Collaboration’s tool for assessing risk of bias)**

|  | Random sequence generation (selection bias) | Allocation Concealment (selection bias) | Blinding of participants and personnel (performance bias) | Blinding of outcome assessment (detection bias) | Incomplete outcome data (short term: ≤ 12 weeks) (attrition bias) | Incomplete outcome data (long term: > 12 weeks) (attrition bias) | Selective reporting (reporting bias) | Other major bias |
| --- | --- | --- | --- | --- | --- | --- | --- | --- |
| Bucher 2010 | Low risk | Unclear | High risk | High risk | Low risk | Low risk | Low risk | Low risk |
| Landon 2004 | N/A | N/A | High risk | High risk | Unclear | Unclear | Low risk | Hawthorne effect in control clinics, crossover effect in control clinics. Some clinics mandated to participate (participation bias) |
| Pyne 2011 | Low risk | Unclear | Low risk | Low risk | Low risk | Low risk | Low risk | Low risk |

Colour coding: Red indicates high risk of bias, yellow indicates unclear risk (inadequate information provided in studies), and green indicates low risk of bias

**Table 2: Risk of Bias – Cohort studies and Time series (The Newcastle-Ottawa Quality Assessment Scale)**

|  | Representativeness of exposed cohort – if truly or somewhat representative of the average adult patient in the community with HIV | Representativeness of non-exposed cohort – if drawn from the same community as the exposed cohort | Ascertainment of exposure – if obtained from a secure record or structured interview | Demonstration that outcome of interest was not present at the start of the study | Comparability – if study controls for important confounding factor(s)* | Assessment of Outcome – if independent blind assessment or record linkage | Follow up – if follow up is adequately long for outcomes of interest (1 year) | Adequacy of follow up – if follow up complete (all patients accounted for), or if subjects lost to follow up unlikely to introduce bias (follow up rate ≥ 80%) or if description provided of those lost |
| --- | --- | --- | --- | --- | --- | --- | --- | --- |
| Brown 2002 | ★ | ★ | ★ | ★ | ★ | ★ | ★ | ★ |
| Shuter 2003 | ★ | ★ | ★ | ★ | ★ | ★ | ★ | ★ |
| Kitahata 2003 | ★ | ★ | ★ | ★ | ★ | ★ | ★ |  |
| Natha 2008 | ★ | ★ | ★ | ★ |  | ★ | ★ | ★ |
| Horswell 2008 | ★ | ★ | ★ | ★ | ★ | ★ | ★ |  |
| Gardner 2008 | ★ | ★ | ★ | ★ | ★ ★ | ★ | ★ | ★ |
| Belperio 2009 | ★ | ★ | ★ | ★ |  | ★ | ★ | ★ |
| Fonquernie 2010 | ★ | ★ | ★ | ★ |  | ★ | ★ |  |
| Ma 2010 | ★ | ★ | ★ | ★ |  | ★ |  | ★ |
| Morris 2009 | ★ | ★ | ★ | ★ |  | ★ | ★ |  |
| Were 2010 | ★ | ★ | ★ | ★ |  | ★ |  |  |
| Youngleson 2010 | ★ | ★ | ★ | ★ |  | ★ | ★ |  |

*Two stars possible for comparability, if the study controls for more than one important variable.

Note: Red indicates that no description for the specified criteria was provided in the study.

Appendix 4: The Evidence Table - Summary of all included studies

| **Record ID**  **Author (year)**  **Country** | **Participants** | **DS/CIS by EPOC taxonomy**  **Control group** | **Measures of Assessment** | **Equity considerations** |
| --- | --- | --- | --- | --- |
| 2486  Brown (2002)  USA | 73 HIV positive pediatric and adolescent patients at one multidisciplinary hospital site, no demographics provided | **CIS - Reminders**  Patient data flowsheet  Control: retrospective chart review before implementation | 1. **Health care process/performance -rates of screening for HIV-related illness** (hepatitis profile): 40% vs. 90% (χ2 = 39.7, p≤0.001) 2. **Health care process/performance -rates of screening for HIV-related illness** (toxoplasmosis IgG Ab titre): 18% to 89%, (χ2 = 67.32 p≤0.001) 3. **Health care process/performance - rates of screening for HIV-related illness** (baseline EKG or ECHO): 43% vs. 67%, (χ2 =  p≤0.01) 4. **Health care process/performance - rates of screening for HIV-related illness** (chest X-Ray): 33% vs. 75%, (χ2 =  p≤0.001) 5. **Health care process/performance - rates of screening for HIV-related illness** (baseline pneumococcal Ab): 23% vs. 91%, (χ2 =  p≤0.001)   All rates unadjusted | **1) Age:** Pediatric and adolescent patients aged 2 weeks to 20 years |
| 3635  Shuter  (2003)  USA | 1026 HIV positive women | **CIS – Audit and feedback**  Weekly list of patients with Pap testing due.  Control: Retrospective collection of baseline data | 1. **Health care process/performance - rates of screening for HIV-related illness** (Pap smear by quarter (Q) compared with preceding quarter): Q0 61.4% vs. Q1 67.4%, p<0.001 (% increase: 6%) vs. Q2 73.7%, p<0.001 (% increase: 6.3%) vs. Q3 74.8%, p=0.37 vs. Q4 73.0%, p=0.13. The OR of a given visit being associated with an up-to-date Pap smear after the intervention as compared to before the intervention was 1.72 (95% CI 1.53± 1.93, *P*<0.001). | 1. **Age:** Mean age 42.4± 9.5 years 2. **Gender:** all female 3. **Race/culture/ethnicity:** Latino 491/940 (52.2%), Black 403/940 (42.9%), White 42/940 (4.5%) 4. **Transmission risk group:** Heterosexual 653/939 (69.5%), IVDU 132/939 (14.1%) |
| 3854  Landon (2004)  USA | 44 intervention clinics and 25 control clinics  - Clinics had to have at least 100 HIV positive cases per year  - 6406 intervention (3190 pre 3216 post) clinics and 3580 control (1761 pre 1819 post), average patients per centre 72 (37-77) | **CIS – Presence and organisation of quality monitoring mechanisms**   Quality improvement collaborative – selected population of focus (HIV), worked collaboratively using Plan-Do-Study-Act cycle to improve HIV care, involved kick off session and 3 learning sessions over 16 month period to allow implementation, monthly reports were submitted and chart reviews performed of key quality of care indicators  Control: 25 control clinics matched by location (urban/rural) region, size, and clinic type | 1. **Immunological or virological** (% difference before/after in VL < 400): 11% vs. 5.4% in controls, p=0.18 2. **Health care process/performance – proportion of patients on ART** (at last visit): % difference before/after: increased CD4 count -3.0 vs. +2.9, p>0.2 3. **Health care process/performance - proportion of patients on indicated prophylaxis** (PCP, % difference before/after):0.4 vs. 3.5, p>0.2 4. **Health care process/performance - rates of screening, for HIV-related illness** (TB, % difference before/after): 0.1 vs. -2.1, p>0.2 5. **Health care process/performance - rates of screening, for HIV-related illness** (pap smear, % difference before/after): 4.6 vs. -4.2, p=0.06 6. **Health care process/performance - rates of appropriate vaccination** (influenza, % difference before/after): 7.3 vs. 6.8, p>0.2 7. **Health care process/performance - rates of screening, for HIV-related illness** (Hepatitis C, % difference before/after): 5.5 vs. 6.2, p>0.2 8. **Economic – information about healthcare utilization** (visits to clinic provider in 3 or 4 quarters (increased perceived to be improvement in care), % difference before/after): 5.4 vs. 2.7, p>0.2   All rates are adjusted for patient age, sex, HIV-related diagnoses, comorbid conditions, and lowest CD4 count over the review period as well as clinic location, region, type, site size, and specialty status.  No differences between new and ongoing clinics, or for those who were ‘poor performers’ at baseline | 1. **Age**: approximately age 40 in both groups 2. **Gender**: 2/3 male in both groups 3. **Transmission risk group**: Substance abuse: Before 15.7% in intervention group, 18.0% in control; after 14.1% in intervention group, 18.2% in control group |
| 3872  Kitahata (2003)  USA | 1204 HIV positive patients >18 years | **CIS – Reminders** Electronic clinical reminder system for prevention of opportunistic infections (OIs) and ART based on guidelines  Control: same population pre-intervention | 1. **Health care performance/process - rates of screening for HIV-related illness** (proportion of those with CD4 ≥ 350 cells/mm^3^   receiving CD4 testing at least every 6 months): 96% vs. 97%   1. **Health care performance/process - rates of screening for HIV-related illness** (proportion of those with CD4 < 350 cells/mm^3^   receiving CD4 testing at least every 3-4 months): 87% vs. 91%   1. **Health care performance/process - rates of screening for HIV-related illness** (proportion of those with CD4 ≥ 350 cells/mm^3^   receiving VL testing at least every 6 months): 96% vs. 97%   1. **Health care performance/process - rates of screening for HIV-related illness** (proportion of those with CD4 < 350 cells/mm^3^ receiving CD4 testing at least every 3-4 months): 93% vs. 91% 2. **Health care process/performance - proportion of patients on antiretroviral therapy**: 72% vs. 81% 3. **Health care process/performance - proportion of patients on indicated prophylaxis** (PCP): 72%vs. 81%, adjusted HR 1.16 (0.84-1.59), p>0.2 4. **Health care process/performance - proportion of patients on indicated prophylaxis** (MAC): 21% vs. 49%, adjusted HR 3.84 (1.58-9.32, p=0.003) 5. **Health care process/performance - rates of screening for HIV-related illness** (TB): 41% v 43%, adjusted OR not provided, p>0.2 6. **Health care process/performance - rates of screening for HIV-related illness** (pap smear): 54% vs. 71%, adjusted OR 2.09 (1.04-4.16), p=0.4 7. **Health care process/performance - rates of screening, for HIV-related illness** (Toxoplasmosis gondi within 90 days): adjusted OR 1.86 (1.05-3.27, p=0.03) 8. **Health care process/performance - rates of screening for HIV-related illness** (initial screening for syphilis): P>0.2, no difference in adjusted odds 9. **Health care process/performance - rates of screening for HIV-related illness** (repeat syphilis after previous negative): adjusted OR 3.71 (2.37-5.81, p<0.0001) | 1. **Age:** 30-39y 49.5% 2. **Gender:** male 87% 3. **Race/ethnicity culture** white 56.2%, Black 16.6% 4. **Transmission risk group:** MSM 56.2%, IDU 23.5%, heterosexual 13.4%, 23% of those receiving appropriate ART, 23% were IVDU vs. 38% of those not receiving ART 5. **Socioeconomic status:** publicly insured 77.2%   No significant difference in prophylaxis/screening and screening by sex, race, transmission group |
| 5654  Natha (2008)  UK | 100 HIV positive adults | **DS - explicit mention of implementation of guidelines into practice**  Use of a proforma (template) for first visit, previously piloted, including discussion prompts  - was validated in consultation and in small pilot  - 18 items for men and 20 for women  Control: Compared pre-proforma (randomly selected and reviewed 50 charts from Oct 04 to Oct 05) with post-proforma (randomly selected and reviewed 50 charts from Oct 05 to Oct 06) | 1. **Health care process/performance** (documentation of HIV risk factors): 62.2% vs. 100%, p0.0001 2. **Health care process/performance - provision of counseling** (discussion re safe sex): 24.4% vs. 65.1%, p = 0.0001 3. **Health care process/performance - rates of screening for HIV-related illness** (recording cervical cytology): 60% vs. 73.3%, p=0.39 4. **Health care process/performance - rates of screening for HIV-related illness** (recording hepatitis serology/vaccine): 62.2% vs. 97.7%, p=0.0001 5. **Health care process/performance - rates of screening for HIV-related illness** (recording STI screen): 36.7% vs. 69.8%, p=0.0009 | 1. G**ender:** pre-intervention 50% male, post-intervention 70% male |
| 6151, Horswell (2008) USA | 3708 HIV positive patients | Disease management system (multidisease programs, including HIV)  **CIS – Change in medical records**  **CIS – Reminders** – Providers received patient level summary of risk factors and disease management related information  Control: first year of program implementation  Part of large multiintervention and multicondition program, interventions adopted differently at different clinical sites | 1. **Economic - information about healthcare utilization**: (visit in last 3 months, goal is to increase) (0.65 vs. 0.66, NS) 2. **Medical – hospitalizations** (Hospital admission rate (/1000 patients over 3 months): 79 vs. 69, NS 3. **Health care process/performance - proportion of patients on indicated prophylaxis** (PCP): 0.73 vs. 0,76, NS 4. **Health care process/performance - proportion of patients on indicated prophylaxis** (MAC): 0.6 vs. 0.66, NS 5. **Health care process/performance - rates of screening for HIV-related illness** (% with viral testing in past 6 months): 0.62 vs. 0.59, NS 6. **Health care process/performance - rates of screening for HIV-related illness** (% with CD4 testing in past 6 months): 0.66 vs. 0.6, NS 7. **Immunological/viral (**% with VL undetectable) 0.37 vs. 0.50, p<0.05 8. **Immunological/viral** (% with CD4 level >200): 0.74 vs. 0.75, NS 9. **Health care process/performance - rates of screening for HIV-related illness** (pap smear within last year): 0.02 vs. 0.39, p<0.05 10. **Health care process/performance- proportion of patients on antiretrovirals** (% with CD4<200 on ART): 0.49 vs. 0.68, p<0.05   Rates adjusted for age, race, gender (if applicable) | 1. **Age:** results adjusted for age (mean not reported) 2. **Gender**: results adjusted for gender (% not reported) 3. **Race/ethnicity/culture**: rates adjusted for race (categories not reported) 4. **Socioeconomic:** also analyzed data based on uninsured status - uninsured patient analysis showed P<0.05 (significant) for outcomes 4), 6), 7), 9), 10) 5. **Socioeconomic:** Publicly insured 67% |
| 6745  Gardner (2008)  USA | 1091 HIV positive patients > 18 years  Cohort recruitment unclear  Only 70% completed all 3 waves with no difference between those who completed all vs. some questionnaires | **DS –Educational meetings**  Provider training to deliver a behavioral intervention regarding communications skills and counseling regarding safer sex/drug use to all patients, had a 4 hour training session, total 182 providers (17-52 per clinic), booster training at 1-2 months into the intervention  **CIS – Audit and feedback**  In one clinic with electronic medical records (EMR) if rates of counseling dropped below 70% they were encouraged to improve this aspect of care  Control: responses from same population at baseline | Primary:   1. **Medical - change in at-risk behaviors** (occurrence of anal or vaginal intercourse (UAVI) without a condom in the past 3 months): unadjusted T0 42%, T1 26%, T2 follow-up 23%, β =0.43, SE=0.05, trend p<0.001, adjusted UAVI β =0.46, SE=0.05, trend p<0.0001.   Secondary:   1. **Health care process/performance: identification of at-risk behaviors** (patient report that provider had discussed safer sex at one or more clinic visits): unadjusted T1 89%, T2 96%   There was a dose response relationship between UAVI and safer sex counseling; those reporting counseling at all, some or no visits had relative reductions from baseline of 44.7% (p<0.001), 34.5% (p<0.0001) and 19.4% (p = 0.22). | Included patients had to be English speaking   1. **Age:** 43.9% 35-45yrs. UAVI more likely in younger than 35 vs. older than 45. 2. **Race/ethnicity/culture:** Black 445/767 (58.0%), White, Non-Hispanic 213/767 (27.8%), Hispanic 77/767 (10%). The significant reduction of UAVI was maintained across all race/ethnicity except “other”. 3. **Transmission risk group**: MSM 46.5%, heterosexual women 31.7%. The significant reduction of UAVI was maintained across all transmission risk groups. UAVI was more likely between MSM and heterosexual women than heterosexual men. |
| 9152, Belperio 2009, USA | 7220 HIV positive adults | **DS –Distribution of educational material**s  Providers received clinic specific guidelines for prescribing certain antiretroviral medications  Control: pre-dissemination of prescribing criteria | 1. **Health care process/performance - health care professional adherence to guidelines** (Drug 1 Atazanavir): 66.3% vs. 66.3% vs. 72.9% p<0.001 (early vs. late p= p<0.001, pre vs. post p=0.010) 2. **Health care process/performance - health care professional adherence to guidelines** (Drug 2 Darunavir): 72.6% vs. 78.8% vs. 62.5% p=0.002 (early vs. late p <0.001, pre vs. post p=0.585) 3. **Health care process/performance - health care professional adherence to guidelines** (Drug 3 Enfuviritide): 92.9% vs. 94.8%, NA, (no p), early vs. late p=0.373 4. **Health care process/performance: health care professional adherence to guidelines** (Drug 4 Tipranavir): 78.1% vs. 78.4% vs. 72.0% p0.420, early vs. late 0.214, pre vs. post 0.675 | No equity indicators reported |
| 9228 Bucher (2010) Switzerland | Non-pregnant adult patients on ART  57 intervention physicians seeing 1634 eligible patients who had primary outcomes recorded, 60 control physicians seeing 1632 patients with primary outcomes recorded | **CIS – audit and feedback**  Physicians were provided a patient specific coronary heart disease (CHD) risk profile at clinic visits  Control: both groups received guidelines for CHD risk factor management, control group also received individual profiles | Primary:   1. **Health care process/performance - rates of screening, for HIV-related illness** (total cholesterol mean difference, mmol/L): unadjusted -0.01(CI -0.08-0.07), adjusted -0.02(CI -0.09-0.06)   Secondary:   1. **Health care process/performance - rates of screening for HIV-related illness** (systolic BP mean difference, mmHg): unadjusted: -0.5 (CI -1.7-0.8), adjusted -0.4 (CI -1.6-0.8) 2. **Health care process/performance - rates of screening for HIV-related illness** (diastolic BP mean difference, mmHg) unadjusted -0.5 (CI -1.6-0.6), adjusted -0.4 (CI-1.5-0.7) 3. **Health care process/performance - rates of screening for HIV-related illness** (Framingham risk score mean difference) unadjusted -0.2% (CI-0.5-0.1), adjusted -0.2% (CI-0.5-0.1) | 1. **Age:** Mean age in both groups was 44 years. Results adjusted for age 2. **Gender:** groups had 71% and 70% males. Results adjusted by gender. 3. **Transmission risk group:** groups had 19% vs. 21% IDU. Results were adjusted by mode of transmission. |
| 9529 Fonquernie  (2010)  France | 1717 HIV positive adults | **CIS – Change medical record**  Use of computer system to track patients over time  **CIS - Reminders**  Alerts for cardiovascular risk, use of track sheets for pap smears  **DS – Explicit implementation of guidelines**  Use of questionnaires to identify key conditions  Control: standard of care pre-intervention | 1. **Medical – adherence to medication** (ART): 72% vs. 81% p<0.001 2. **Health care process/performance - rates of screening for HIV-related illness** (cardiovascular risk as measured by number of risk factors (RF)): 0RF, 22% vs. 24%, 1RF 40% vs. 39%, 2RF 26% vs. 26%, ≥3RF 12% vs. 11%, p<0.027 3. **Health care process/performance - rates of screening for HIV-related illness** (glycemia ≥6.1mmol/L): 13.9% vs. 7.6%, p=0.08 4. **Health care process/performance - rates of screening for HIV-related illness** (BP systolic >140mmHg or diastolic >90mmHg): 7.5% vs. 8.2%, ns 5. **Health care process/performance - rates of screening for HIV-related illness** (HDL <1mmol/L): 31.4% vs. 24.6%, p<0.001 6. **Health care process/performance - rates of screening for HIV-related illness** (LDL >4.1 mmol/L): 14.5 vs. 10.2%, p<0.001 7. **Health care process/performance - rates of screening for HIV-related illness** (present tobacco use): 34.0% vs. 35.8%, ns 8. **Health care process/performance - rates of screening for HIV-related illness** (Pap smears): 44% vs. 51.4%, p=0.04 9. **Health care process/performance - rates of screening for HIV-related illness** (identified as requiring Hepatitis B immunization): 16.5% vs. 17%, ns 10. **Psychosocial** (proportion of patients receiving measure of sexual dysfunction): 7% vs. 27%, P<0.001   Outcomes are unadjusted | 1. **Age:** mean age 42.9 +-9.8y 2. **Gender:** male 75.0% 3. **Race/ethnicity/culture:** 64.8% French origin and 19.3% Sub-Saharan Africa 4. **Transmission risk group**: 82.6% identified sexual intercourse as transmission risk group (no further details); IVDU 142/1946 (7.3%), sexual intercourse 1589/1946 (81.6%), hemophiliacs and transfused after 1978 31/1946 (1.6%), other 48/1946 (2.5%) |
| 10008  Ma  (2010)  USA | 263 patients in the clinic, 100 received recommendations, 75 included in intervention | **DS - Communication and case discussion between distant health professionals** – Presence of clinical pharmacist aiming to optimize ART regimens through discussion with prescribing provider  Control: 6 month period before change in ARV regimen for each patient | 1. **Immunological/virological** (mean CD4 count, cells/mm^3^): 462 vs. 491, p=0.13 2. **Immunological/virological** (mean CD4 %): 23% vs. 25%, p=0.007 3. **Immunological/virological**(% with VL<75 copies/mL): 63% vs. 96%, P<0.0001 4. **Medical - adherence to medication** (% adherence to medication based on pharmacy refills): 81% vs. 89%, p=0.003 | 1. **Age:** mean age 49.32 years (30-73) 2. **Gender:** 78.7% male 3. **Race/ethnicity/culture:** White 45/75 (60%), Black 19/75 (25.3%), Hispanic 7/75 (9.3%) |
| 10026  Magnus, (2009) USA | Participation is at level of provider: 196 providers responded in total, T0=105, T1=46, T2=45  80% female, 68.3% age 30-50  31.2% nurses and 28.7% MD | **CIS – Reminders**  **CIS - Changes in medical records systems**  Implementation of an electronic clinical management system to supplement not replace paper  Control: T0 (preimplementation) | 1. **Health care process/performance** (highlighting 3 questions relating to provider satisfaction in survey instrument): 2. “I am satisfied with the systems in place at this clinic to track patient information”: T0 1.2%, T1 2.8% T2 15.8%, P<0.05, when adjusted by provider, physicians were less likely that others to agree (OR 0.42, 0.21-0.80, while this did increase over time OR 1.62, 1.09-2.40) 3. “LabTracker has facilitated my clinical decision-making”: T0 n/a T1 7.7% T2 17.1%, ns, when adjusted by provider, physicians were less likely that others to agree (OR 0.33, 0.12-0.97) while those who used the system once per day or more were more likely than others to agree OR 3.38, 1.38-8.28) 4. “It is easier to find CD4 counts in the paper medical chart than in LabTracker”: T0 n/a T1 9.4% T2 13.5%, ns, when adjusted by provider those who used the system once per day or more were less likely than others to agree OR 0.33, (0.14-80.75) | No patient level equity indicators provided |
| 10162, Morris  (2009) Zambia | Health providers in Zambia: 516 adult HIV, 270 pediatric HIV, 341 adherence counseling, 91 specialty nurse triage, 93 intensive clinical mentorship | **DS - Educational meetings**  Didactic traineeships aimed to task-shift clinical responsibilities (e.g. doctor duties to clinical officers) and short term clinical mentoring  **CIS – Presence and organisation of quality**  **monitoring mechanisms**  **CIS – Audit and feedback**  Control: pre-implementation period | 1. **Health care process/performance - rates of screening for HIV-related illness** (% with baseline ALT, shown graphically): approx. 50% to approx. 78% 2. **Health care process/performance - rates of screening for HIV-related illness** (% with baseline Hb, shown graphically): approx. 65% to approx. 82% 3. **Health care process/performance -proportion of patients on appropriate prophylaxis** (PCP, shown graphically): approx. 28% to approx. 78%   Number of denominator patients unknown, approximately 1000 in May 2004 to 72000 in November 2007  Used June 2005 and December 2007 data for approximate values above | No equity indicators provided, no adjustment |
| 10398 Pyne, (2011)  USA | HIV positive patients with scores of 10 or higher on PHQ-9 depression scale, not suicidal or cognitively impaired and had access to a telephone | **DS - Communication and case discussion between distant health professionals** Establishment of a HIV depression care team (depression care manager, clinical pharmacist, psychiatrist) who communicated with clinicians via EMR and supported patients via telephone  Control: Both intervention and control received DS - Distribution of educational materials in the form of depression scales for patients to complete prior to clinical encounters, but the control group did not have the involvement of the HIV depression care team | Primary:   1. **Psychosocial** (mean depression severity score (0-4) by SCL-20): NS difference at 6 and 12 months 2. **Psychosocial**  (depression treatment response 50% or greater in mean SCL-20 score compared to baseline): at 6 months unadjusted OR 2.50 (1.37-4.56) adjusted OR 2.60 (1.39-4.86), at 12 months unadjusted OR 1.37 (0.78-2.41) adjusted 1.29 (0.72-2.32) 3. **Psychosocial** (depression remission mean SCL-20 score less than 0.5): at 6 months unadjusted OR 2.25 (1.11-4.54) adjusted OR 2.40 (1.10-5.22), at 12 months unadjusted OR 1.52 (0.78-2.98) adjusted 1.36 (0.66-2.88) 4. **Psychosocial – health related quality of life** (QWB-SA score (0.0-1.0)): NS   Secondary:   1. **Medical – health status** (physical and mental health component summary scores from the Medical Outcomes Study Beterans 12-Item Short-Form Health survey): NS 2. **Medical** (change in HIV symptoms 20-item Symptoms Distress Module score): at 6 months unadjusted β = -2.6 (-3.5 to -1.8) adjusted β=-0.62 (-1.16 to -0.08), at 12 months unadjusted β=-0.82 (-1.6 to -0.07), adjusted β=-0.09, -1.58 to 1.4) 3. **Medical – adherence to medications** (antidepressants): NS 4. **Medical – adherence to medications**: (antiretrovirals): NS   The factors controlled for varied by outcome. | 1. **Age**: mean ages by group 49.8y and 49.8y 2. **Gender:** male 97.6% and 96.8% 3. **Race/ethnicity/culture:** African American 78/123 (63.5%) in intervention group and 77/126 (61.6%) in control group 4. **Education:** High school graduate or higher 95.9% and 89.7% 5. **Socioeconomic status:** Annual income ≥ $20,000 50.8% and 42.6%   No significant difference between groups |
| 10874  Were  (2010)  Uganda | 88 patient before, 94 patients after, based on 3 providers before and 3 providers after  No patient level data provided | **CIS – Reminders**  **CIS - Changes in medical records systems**  Computer generated clinical summaries  Alert to perform CD4 count if none in previous 6 months Before implementation of clinical summaries  Control: pre-implementation of intervention | 1. **Economic – Information about health care utilization** (total visit time (min)) 197.7 vs. 186.2, mean difference -11.5, p<0.001 2. **Economic – Information about health care utilization** (time spent in direct patient care (min/visit)) 2.9 vs. 2.3, p <0.001 3. Economic – Information about health care utilization (time spent in indirect patient care (min/visit)) 3.2 vs. 2.9, p =0.7 | No equity indicators provided |
| 10921  Youngleson  (2010)  South Africa | No further patient or provider level information on participants | **CIS - Presence and organisation of quality monitoring mechanisms**  Increased education (q 6month workshops), QI methods education and mentoring, identification of gaps, multidisciplinary improvement teams, identification of change strategies, addition of resources, QI protocol changes | 1. **Health care process/performance - proportion of patients on antiretrovirals** (proportion on antenatal AZT): 74% to 89%, P<0.001 2. **Health care process/performance - proportion of patients on antiretrovirals** (proportion on antenatal HAART): 10% to 25%, P<0.001 3. **Health care process/performance - proportion of patients on antiretrovirals** (proportion of women receiving NVP in labour): 74% to 86%, P<0.001 4. **Health care process/performance - proportion of patients on antiretrovirals** (proportion of women receiving AZT in labour): 43% to 84%, P<0.001 | No equity indicators provided |

**Appendix 5: Equity Indicators in Included Studies**

| **Indicator** | **Number of Studies reporting indicator** | **Additional Comments** |
| --- | --- | --- |
| Age | 9 studies[^29-31^](#_ENREF_29)^,^[^33^](#_ENREF_33)^,^[^35^](#_ENREF_35)^,^[^36^](#_ENREF_36)^,^[^38^](#_ENREF_38)^,^[^39^](#_ENREF_39)^,^[^41^](#_ENREF_41) | The mean age or age range of the highest proportion of participants in all studies was less than 50 years. Four included studies [^31^](#_ENREF_31)^,^[^32^](#_ENREF_32)^,^[^36^](#_ENREF_36)^,^[^38^](#_ENREF_38) adjusted outcome data for age and Kitahata et al. [^41^](#_ENREF_41) describe that outcomes were consistent across age groups. There were no clear patterns of interventions effective for populations by age. |
| Gender | 8 studies[^29^](#_ENREF_29)^,^[^31^](#_ENREF_31)^,^[^35-39^](#_ENREF_35)^,^[^41^](#_ENREF_41) | All but one examining cervical screening[^29^](#_ENREF_29) comprised a sample of mostly male participants. Four [^31^](#_ENREF_31)^,^[^32^](#_ENREF_32)^,^[^36^](#_ENREF_36)^,^[^38^](#_ENREF_38) studies reported adjusting outcome data for sex or gender and Kitahata et al. [^41^](#_ENREF_41) report that outcomes were consistent between male and female participants. |
| Socio-economic status | 3 studies^31,35,40^ | Two studies[^32^](#_ENREF_32)^,^[^41^](#_ENREF_41) reported that most of the participants had public insurance (77.2% and 67%, respectively) but only Horswell et al.[^32^](#_ENREF_32) adjusted their findings for insurance status and found that outcomes of health care process/provider performance were significant more often in those who were uninsured versus those who were not (4/6 vs. 2/6 outcome indicators). One study[^36^](#_ENREF_36) reported that the largest proportion of annual income was in the ≥ $20,000 category (with no other categories provided) and that outcomes were adjusted for socioeconomic status. |
| Age | 1 study^35^ | The study was conducted in the U.S. with 95.9% of participates being high school graduates and 89.7% having higher education. |
| Transmission Risk | 6 studies[^29^](#_ENREF_29)^,^[^31^](#_ENREF_31)^,^[^33^](#_ENREF_33)^,^[^38^](#_ENREF_38)^,^[^39^](#_ENREF_39)^,^[^41^](#_ENREF_41) | Findings were heterogeneous. Intravenous drug use (IVDU)/substance abuse was the most commonly described risk group (5 studies [^29^](#_ENREF_29)^,^[^31^](#_ENREF_31)^,^[^38^](#_ENREF_38)^,^[^39^](#_ENREF_39)^,^[^41^](#_ENREF_41) with IVDU rates ranging from 7.3% to 23.5% of the populations. With respect to sexual risk, 2 studies[^33^](#_ENREF_33)^,^[^41^](#_ENREF_41) reported the highest proportion of patients as men who have sex with men, 1[^29^](#_ENREF_29) reported the highest proportion as heterosexual transmission, and 1[^39^](#_ENREF_39) simply as ‘sexual intercourse’. Only 1 study[^38^](#_ENREF_38) reports adjusting for transmission risk group and 3 studies [^33^](#_ENREF_33)^,^[^36^](#_ENREF_36)^,^[^41^](#_ENREF_41) report that outcomes remained significant across transmission risk groups. |
| Race/Culture/Ethnicity | 7 studies [^29^](#_ENREF_29)^,^[^32^](#_ENREF_32)^,^[^33^](#_ENREF_33)^,^[^35^](#_ENREF_35)^,^[^36^](#_ENREF_36)^,^[^39^](#_ENREF_39)^,^[^41^](#_ENREF_41) | 2 reported the highest proportion of patients as Black or African-American[^33^](#_ENREF_33)^,^[^36^](#_ENREF_36), 2 as White [^35^](#_ENREF_35)^,^[^41^](#_ENREF_41), 1 as French (unspecified) [^39^](#_ENREF_39), and 1 as Latino[^29^](#_ENREF_29). Three studies[^32^](#_ENREF_32)^,^[^36^](#_ENREF_36)^,^[^41^](#_ENREF_41) describe adjusting outcomes for race, culture or ethnicity but 1[^32^](#_ENREF_32) did not report subcategories, and 2 studies[^33^](#_ENREF_33)^,^[^41^](#_ENREF_41) describe that outcomes were consistent across all race/ethnicities reported (except for the category ‘other’ in Gardner [^33^](#_ENREF_33), which comprised 4.2% of the study population). |
